# DNA Methylation and All-Cause Mortality in Middle-Aged and Elderly Danish Twins

**DOI:** 10.3390/genes9020078

**Published:** 2018-02-08

**Authors:** Anne Marie Svane, Mette Soerensen, Jesper Lund, Qihua Tan, Juulia Jylhävä, Yunzhang Wang, Nancy L. Pedersen, Sara Hägg, Birgit Debrabant, Ian J. Deary, Kaare Christensen, Lene Christiansen, Jacob B. Hjelmborg

**Affiliations:** 1 Epidemiology and Biostatistics, Department of Public Health, University of Southern Denmark, 5000 Odense, Denmark; amsvane@health.sdu.dk (A.M.S.); msoerensen@health.sdu.dk (M.S.); jlund@health.sdu.dk (J.L.); qtan@health.sdu.dk (Q.T.); bdebrabant@health.sdu.dk (B.D.); KChristensen@health.sdu.dk (K.C.); lchristiansen@health.sdu.dk (L.C.); 2 Unit of Human Genetics, Department of Clinical Research, University of Southern Denmark, 5000 Odense, Denmark; 3 Department of Medical Epidemiology and Biostatistics, Karolinska Institutet, SE-171 77 Stockholm, Sweden; juulia.jylhava@ki.se (J.J.); yunzhang.wang@ki.se (Y.W.); nancy.pedersen@ki.se (N.L.P.); Sara.Hagg@ki.se (S.H.); 4 Department of Psychology, University of Edinburgh, Edinburgh EH8 9JZ, UK; i.deary@ed.ac.uk; 5 Center for Cognitive Aging and Cognitive Epidemiology, University of Edinburgh, lEdinburgh EH8 9JZ, UK

**Keywords:** DNA methylation, mortality, EWAS, survival analysis, survival prediction, twin study, heritability

## Abstract

Several studies have linked DNA methylation at individual CpG sites to aging and various diseases. Recent studies have also identified single CpGs whose methylation levels are associated with all-cause mortality. In this study, we perform an epigenome-wide study of the association between CpG methylation and mortality in a population of 435 monozygotic twin pairs from three Danish twin studies. The participants were aged 55–90 at the time of blood sampling and were followed for up to 20 years. We validated our results by comparison with results from a British and a Swedish cohort, as well as results from the literature. We identified 2806 CpG sites associated with mortality (false discovery rate (FDR)<0.05), of which 24 had an association *p*-value below $10^{-7}$. This was confirmed by intra-pair comparison controlling for confounding effects. Eight of the 24 top sites could be validated in independent datasets or confirmed by previous studies. For all these eight sites, hypomethylation was associated with poor survival prognosis, and seven showed monozygotic correlations above 35%, indicating a potential moderate to strong heritability, but leaving room for substantial shared or unique environmental effects. We also set up a predictor for mortality using least absolute shrinkage and selection operator (LASSO) regression. The predictor showed good performance on the Danish data under cross-validation, but did not perform very well in independent samples.

## 1. Introduction

Epigenetics have become very important in our understanding of aging processes. DNA methylation is an epigenetic mechanism that affects gene expression, and changes in the methylation pattern have been linked to various diseases [1,2,3,4]. DNA methylation is partly inherited, and partly influenced by environmental and random factors, as well as natural aging.

The degree of DNA methylation at certain CpG sites has been shown to change consistently with age in various organs and in the blood. This has led to the establishment of epigenetic “clocks” that predict chronological age from methylation patterns [5,6]. In fact, several collections of CpG sites for which the methylation level associates very well with age have been identified, suggesting that fundamental dynamical processes are reflected in, or at least associated with, such subsets of sites. Although the functioning of these sites is largely unknown, it is found that they also can be associated with all-cause mortality, i.e., age at death. Interestingly, previous studies have found that the deviation between methylation age as defined in [5,6] and chronological age is associated with all-cause mortality [7,8].

Several recent epigenome-wide association studies (EWAS) have identified individual CpG sites related to all-cause mortality. In [9], 58 CpG sites linked to mortality are reported, and 10 of these are used to construct a mortality risk score that can be validated in independent data. CpG sites connected with mortality in very old people (above 90) are discovered in [10], and in [11] single CpGs are associated with mortality and compared to age-related CpGs. In all three studies, the CpG sites most strongly associated with mortality are different from those typically associated with age.

In this study, we perform EWAS analysis of DNA methylation in blood and survival data for 870 monozygotic (MZ) twins from the Danish Twin Register. Exploiting the fact that twins are naturally matched with respect to genes and early childhood environment, confounding of the association between site-specific methylation and survival time may be reduced by adjusting for twin pairing. To model within-pair dependence, we use a Cox frailty model to assess the influence of individual sites on time to death. Sites selected under false discovery rate criteria are compared to findings in the literature. Furthermore, a mortality predictor is constructed and validated in independent datasets. Random selection of twin pairs from those available allows us to use the twin design to obtain an upper limit for the genetic influence on methylation of mortality-associated CpG sites.

## 2. Materials and Methods

### 2.1. Study Population

The study comprises data on 435 pairs of monozygotic twins from three datasets collected by the Danish Twin Register: a study of extremely birth weight-discordant twins (BWD) [12], the Longitudinal Study of Aging Danish Twins (LSADT) [13], and the Middle Aged Danish Twins Study (MADT) [14]. Blood samples were taken over the periods 1996–1997 for LSADT and 2008–2011 for MADT and BWD. Dates of births and deaths were obtained from the Danish Civil Registration System in 2016. By then, 258 deaths had occurred. Table 1 shows the basic characteristics of the study population.

### 2.2. Methylation Data

DNA was isolated from buffy coats using the salt precipitation method, and bisulfite conversion of 500 ng genomic DNA was performed using the EZ Methylation Gold Kit (Zymo Research, Orange County, CA, USA). The methylation degree at 485,512 CpG sites was analyzed using the Infinium 450K HumanMethylation BeadChip (Illumina, San Diego, CA, USA) following the manufacturer’s instructions at either the Leiden University Medical Center or GenomeScan B.V., Leiden, the Netherlands. BeadChip images were scanned using the iScan system. Twin pairs were analyzed on the same array. The three datasets were analyzed on different occasions.

Data preprocessing and quality control was performed in line with [15] using the R-package MethylAid. Samples not meeting the quality requirements were excluded. Probes with detection *p*-value >0.01, no signal, or bead count <3 were treated as missing. CpG sites with more than 5% missing values, probes targeting sex chromosomes, and cross-reactive probes were excluded from the analysis. Polymorphic sites were kept in the analysis, but polymorphic probes with allele frequency of least 1% in the European population [16] are marked in the results tables below. Data was normalized using functional normalization [17] with four principal components. After preprocessing, a total of 441,160 CpG sites remained for further analysis.

Blood cell type composition was measured for most MADT and BWD participants and this was used for imputing the proportion of basophils, eosinophils, monocytes, neutrophils, and lymphocytes in the remaining individuals (see [18] for details). The methylation beta values were corrected for batch effects and cell types using a linear mixed model with sex, age, dataset, and cell type proportions as fixed effects and with sample plate and plate position as random effects. Residuals were used in all subsequent analyses.

### 2.3. Validation Studies

For validation, we used results from two studies. The first one was an EWAS of CpGs associated with mortality [19]. The study population was made up of the Lothian Birth Cohorts LBC1921 and LBC1936 [20], consisting of individuals born in 1921 (N=550) and 1936 (N=1091), respectively. Blood samples were taken in 1999 and 2004, and vital status obtained in 2013 (454 deaths) and 2011 (186 deaths), respectively. Descriptions of the cohorts and methylation data can be found in [7]. Adjustment for cell type composition was performed using the R-package celltypes450.

The second study was from the Swedish Adoption/Twin Study of Aging (SATSA) [21] and included methylation and survival data on 385 individuals aged 48–99 at baseline, of which 231 died before follow-up (up to 20 years). The sample included 73 monozygotic and 96 dizygotic twin pairs. The cell type composition was estimated using the Houseman method [22] and correction for batch effects was done using the ComBat function from the R-package sva [23].

### 2.4. Statistical Analysis

All analyses below used methylation β values corrected for batch effects and cell type composition, and were standardized to a standard deviation of one. In the analyses of the Danish twins, we adjusted for the three different cohorts.

#### 2.4.1. Univariate Analysis

The association between methylation of individual CpG sites and time from blood sample to death was analyzed in the Danish twin data using a Cox frailty model allowing each twin pair to have a unique gamma-distributed hazard ratio modeling pair-specific weaknesses (e.g., genetic or environmental). The model was adjusted for sex, age, and cohort, and the results were corrected for multiple testing by the Benjamini–Hochberg procedure. The Cox proportional hazards assumption was assessed using martingale and Shoenfeld residuals. For CpG sites identified by this procedure, a matched intra-pair comparison of the twins was performed using a stratified Cox model (with pair-specific baseline hazards) in order to control for confounding effects.

#### 2.4.2. Construction of the Predictor

To construct a predictor for survival, we applied the least absolute shrinkage and selection operator (LASSO) in a Cox model using the glmnet package in R in order to select a smaller set of CpG sites that predict survival. The LASSO was forced to include sex, age, and cohort in the model, while the twin structure was ignored for this analysis.

Since the LASSO requires complete observations for all sites and each CpG had a small number of missing values (0.78 missing on average), we used a single imputation, where each missing value was replaced by its expected value in a linear regression of methylation degree on sex, age, cohort, and three principal components.

To make the LASSO output less sensitive to the specific choice of sample, we applied stability selection [24] modified as suggested in [25]. Thus, we ran 1000 replications of the LASSO on randomly chosen subsets of our sample with half the sample size. In each replication, the smoothness parameter was chosen as the minimizer of the partial likelihood under 10-fold cross-validation plus one half standard error. This was an ad hoc choice made to ensure a reasonably sized predictor. The CpGs selected in more than 80% of the replications were chosen for the final model.

Fitting a Cox model with sex, age, and the sites chosen by the LASSO, we used the log hazard ratio as a predictor for mortality. The estimated coefficients were used for predictions in the two validation datasets.

#### 2.4.3. Heritability

To get an upper bound on the heritability of DNA methylation for the CpG sites found in the analyses above, we computed monozygotic twin correlations of methylation levels after correction for sex, age, and cohort.

#### 2.4.4. Validation

The sites discovered in the univariate analysis were validated by fitting a Cox model for each on The Lothian Birth Cohort, LBC, and The Swedish Twin Study of Aging, SATSA, datasets adjusting for sex, age, and twin structure (SATSA). The resulting *p*-values and hazard ratios were compared to those from the Danish dataset. As further validation, we computed a 10-fold cross-validated Harrell’s C [26,27] for a Cox model with sex, age, and each CpG site as covariates. This was done both for the Danish, British, and Swedish datasets and compared to a cross-validated Harrell’s C for the basic Cox model based on sex and age only. A similar validation was performed for the sites involved in the predictor.

To validate the predictor, an overall Harrell’s C for the Cox model with the predictor as the only covariate was computed for all three datasets. Moreover, to investigate whether the effect of the predictor was independent of underlying genetic effects, we conducted an intrapair analysis on the Danish and Swedish twin datasets by fitting a Cox model stratified by the twin pair with the predictor as only covariate. The predictive ability over time of the sites chosen for the predictor was assessed by computing a time-varying area under the ROC curve, AUC, using the timeROC package in R. The AUC was validated by 5-fold cross-validation on the Danish dataset, and rough confidence intervals were constructed by averaging the confidence intervals produced by each fold. Moreover, we computed a time-varying AUC for the predictor on the two validation datasets. Finally, we drew a correlation plot for the methylation levels of the CpG sites chosen for the predictor.

#### 2.4.5. Comparison to the Literature

The sites identified in this study were compared to the CpG sites linked to mortality in previous studies [9,10,11] and the CpG sites associated with aging in [5,6]. A comparison to the EWAS performed on LBC in [19] can be found in that paper.

## 3. Results

We analyzed survival data for 435 monozygotic twin pairs from three Danish twin studies (LSADT, MADT, and BWD). MADT was the largest study with 482 individuals, while the BWD and LSADT included 150 and 238 individuals, respectively. The participants in the LSADT were generally older (73–90 years) and had longer follow-up times (up to 20 years) compared to those of the MADT and BWD (age 55–79, max 8 years of follow-up); thus the most deaths occurred in the LSADT. In total, 258 participants died before the end of the study. MADT and BWD were homogeneous in gender, while 76% of the LSADT participants were female. The basic characteristics of the study population are shown in Table 1. DNA methylation levels were measured for all individuals. After pre-processing, methylation data for 441,160 CpG sites was available.

### 3.1. Univariate Analyses

The association between methylation degree at each single CpG site and survival was analyzed by Cox regression adjusting for sex, age, and cohort while taking the twin pairing into account. This resulted in a total of 2806 CpG sites with false discovery rate (FDR) below 0.05. A full list can be found in Supplementary Table S1. For 2074 of the 2806 top sites (73.9%), hypomethylation was associated with increased mortality corresponding to a hazard ratio (HR) below 1, while hypermethylation was associated with mortality for the remaining 732 sites (HR>1). Twenty-four CpG sites had an unadjusted *p*-value below $10^{-7}$, corresponding to a Bonferroni corrected *p*-value below 0.044. These are listed in Table 2. In a matched intra-pair comparison of the twin and co-twin the suggested direction of association was confirmed for all 24 sites and in 16 of the sites the association was significant at a 0.05 level, as can be seen from Table 2.

For each of the 24 top CpG sites, Table 3 shows the results of a similar Cox analysis on the validation datasets. Comparing the *p*-values and direction of the hazard ratios, seven CpGs could be validated in the Lothian Birth Cohort, namely cg07626482, cg05339037, cg10589813, cg17087741, cg11339912, cg15871086, and cg02711608. For all seven sites, hypomethylation was associated with mortality. In the Swedish dataset, 5 of the 24 CpGs were missing. The remaining 19 sites did not show any convincing effect when multiple testing was taken into account. However, the three lowest *p*-values belong to three of the seven sites validated in the LBC (cg07626482, cg05339037, and cg10589813).

**Figure 1:**
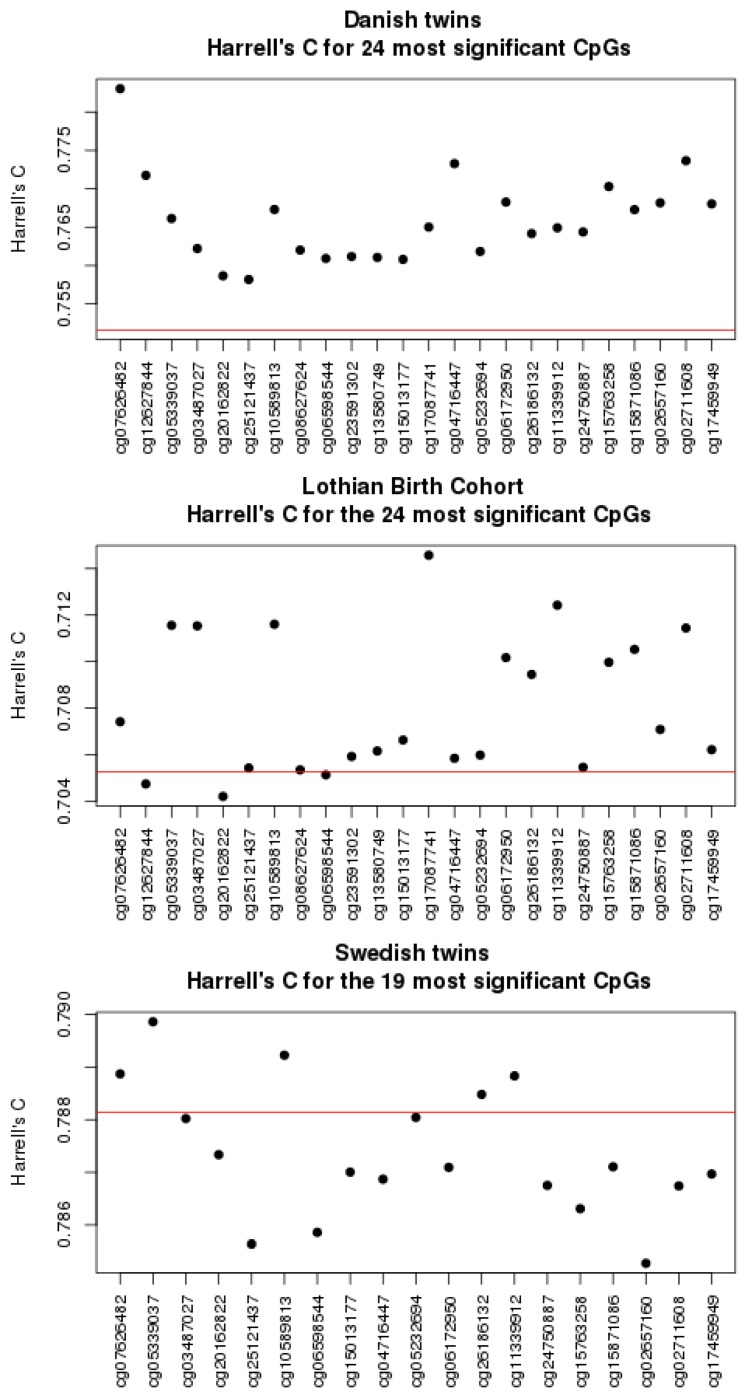
The 10-fold cross-validated Harrell’s C for the 24 most significant sites in the univariate analysis. Sex, age, and cohort are included in the model. The red line corresponds to the model based on sex, age, and cohort only.

As a second validation, we computed a cross-validated Harrell’s C for the Cox model including sex, age, and each of the 24 top sites. Harrell’s C measures the concordance between survival time and the hazard ratio computed from sex, age, and methylation degree. The result is shown in Figure 1 and compared to the model with sex and age only. For the Danish twin data, each CpG site improves the basic model. Most sites also improve concordance in the Lothian Birth Cohort although the magnitude of improvement is smaller. The best validated sites are the seven sites also validated by *p*-values together with cg06172950 and cg15763258 (excluding sites with inconsistent hazard ratio). The 19 sites available in SATSA generally show a poor performance. The only three sites showing moderately good performance are the same as the three best ones based on *p*-values.

There were no obvious violations of the proportional hazards assumption, but some outliers in the methylation data may have influenced the results. An intrapair analysis was not feasible due to sparse information from discordant pairs.

### 3.2. Mortality Predictor

We used LASSO regression to choose 14 CpG sites for a mortality predictor. These sites are listed in Table 4. The predictor is defined as a linear combination of sex, age, and methylation values at the 14 sites with the coefficients given in Table 4.

The individual associations of the 14 sites with mortality were validated using Harrell’s C exactly as in the univariate analysis (see Supplementary Figure S1). They again performed well on the Danish twin data. However, we remark that stability selection serves as a sort of cross-validation in the selection process, and hence the resulting sites can be expected to perform well under cross-validation. Most of the 14 sites could be validated on the LBC data, while the SATSA data again showed poor concordance with survival for the 12 sites available in the dataset. The LASSO tended to choose sites that are uncorrelated. This was confirmed by Supplementary Figure S2, showing negligible correlations between most of the 14 sites. The only exceptions are cg07626482 and cg22304262, which are both located at the gene *SLC1A5*.

As an overall assessment of the predictive ability of the mortality predictor, we computed a Harrell’s C on the Danish twin data. This yielded C=0.85 both with and without 10-fold cross-validation, which was an improvement compared to the model based on sex and age only (C=0.75). However, for the LBC and SATSA datasets, we obtained C=0.69 and C=0.75, respectively, which was in fact worse than the model with sex and age only (C=0.70 and C=0.78, respectively).

**Figure 2:**
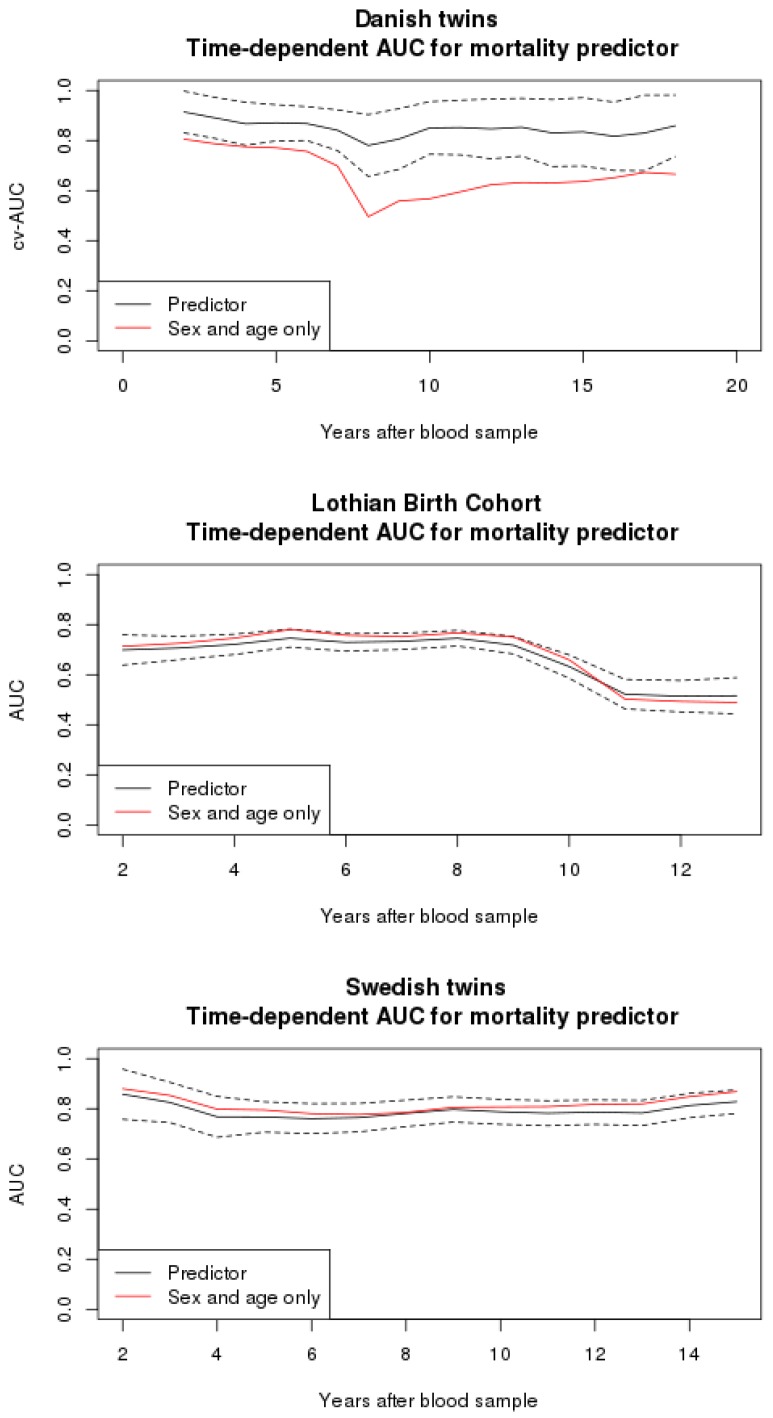
The 10-fold cross-validated time-varying AUC for the LASSO predictor based on all 14 CpG sites, sex, and age. This is compared to the model based on sex and age only.

Figure 2 shows a time-varying AUC measuring the ability of the predictor to predict survival a given number of years after the initial blood sample. It shows a good and time-stable performance in the Danish dataset and is clearly better than the predictor based on sex and age only at all time points. However, the validations on LBC and SATSA do not show any improvement of the standard predictor based on sex and age.

To confirm that the observed effect of the predictor on the Danish data was independent of underlying genetic variation, we conducted an intrapair analysis measuring the predictive ability within twin pairs. We found a highly significant effect of thelpredictor ($p=2.91\;\cdot \;10^{-8}$). Since monozygotic twins are matched on the underlying genes, this shows that the effect of the predictor cannot be due to underlying genetic effects alone. A similar analysis on the SATSA data only obtained p=0.097.

### 3.3. Heritability

The last columns of Table 2 and Table 4 show the correlation between methylation measurements within monozygotic twin pairs for the top 24 sites and the 14 sites in the predictor, respectively, providing an upper bound on the heritability. The correlation highly varies between different CpGs. For some sites, the correlation is essentially 0, while for others, the correlation is as high as 0.53, indicating some heritability or shared environmental effects. It is worth noting that the seven best validated sites in top 24 all have rather high correlations (0.36–0.45) allowing for a potentially high heritability of the mortality-related methylation pattern.

### 3.4. Comparison to Other Studies

In [9], a Cox analysis of data from the Germanl ESTHER study was used to identify 11,063 mortality-related CpG sites (FDR≤0.05). These were boiled down to 58 using LASSO regression in a validation dataset. Two of the top 24 sites from our univariate analysis overlapped with these 58, namely cg07626482 and cg02657160, having the 1st and 22nd lowest *p*-values in our study, respectively. Out of the 58 sites, 19 (33%) had FDR≤0.05 in our study, and 35 (60%) had an unadjusted *p*-value below 0.05 in our study. Ten sites were selected for a final mortality predicting model in [9], of which seven (70%) had p≤0.05. A list of the 35 sites with p≤0.05 in our study can be found in Supplementary Table S2. For all 35 sites, the hazard ratios were consistent in the two studies, and in 31 cases (89%) hypomethylation was associated with mortality.

Data from the Finnish Vitality 90+ study was analyzed in [10]. With 2.55 years of follow-up, 19 mortality-related sites were found (FDR≤0.5). None of these overlapped with our 2806 sites and only one (5.2%) had p≤0.05 (cg08596308, p=0.021). The average *p*-value for the 19 sites was 0.55. Thus, the *p*-values are not lower than what would be expected by chance. With 4-year follow-up data, seven sites with FDR≤0.5 were found in [10]. Again, only cg08596308 reached significance in our study.

In [11], data from the Italian InCHIANTI study was analyzed. Methylation at 88 sites associated with mortality (p≤0.001) was listed in the paper. One of these had FDR≤0.05 (cg00522231, HR=1.34, $p=2.5\;\cdot \;10^{-4}$) in our study, while 21 (24%) had p≤0.05.

Horvath’s methylation age [5] was based on methylation of 353 CpG sites. One of these had FDR≤0.05 in our study (cg01485645, $p=1.4\;\cdot \;10^{-5}$). In total, 35 of the sites (10%) showed significance. The methylation age defined by Hannum [6] is based on 71 CpG sites. None of these are among our top 2806 sites, while 12 (17%) have p≤0.05.

## 4. Discussion

We analyzed DNA methylation data on 870 monozygotic twins to find CpG sites linked to mortality. We found 2806 CpG sites associated with mortality (FDR≤0.05) indicating an abundance of mortality-related CpGs that is supported by [9,19]. We identified 24 sites with a *p*-value less than $10^{-7}$, and out of these, cg07626482, cg05339037, cg10589813, cg17087741, cg11339912, cg15871086, and cg02711608 were validated in an independent dataset, while cg02657160 had been reported in an earlier mortality study [9]. For all eight sites, hypomethylation was associated with increased mortality, and for the first seven sites, MZ correlation ranged from 0.36 to 0.45, indicating an inherited or shared environmental contribution to the methylation of mortality-related sites. The seven best validated sites in the top 24 all had rather high correlations, allowing for a potentially high heritability of mortality-related methylation patterns that is novel and should be pursued in further studies.

In order to reduce the influence of confounders, we compared MZ twins to their co-twin. This basically enables us to study to which degree the twin with the highest methylation value is also the twin in the pair with longest survival. This is a very strong test for association. For all 24 identified, the suggested direction of the CpG survival association was confirmed, and for 16 sites, the *p*-value was below 0.05.

The most significant CpG site in the univariate analysis was cg07626482, which also had the most extreme hazard ratio among the top 2806 sites and the best cross-validated Harrell’s C among the top 24 sites. Moreover, it was chosen by the LASSO in all 1000 replications of the stability selection. This was confirmed in the Lothian Birth Cohort and supported by the Swedish twin data. Interestingly, it was also among the 58 sites reported in [9]. This CpG site is located at the shore of a CpG Island in the 5’ region of the *SLC1A5* gene. The surrounding region contains an enrichment of histone marks and transcription factor binding sites, which may suggest a regulatory role for this region. The *SLC1A5* gene encodes a sodium-dependent neutral amino acid transporter [28]. High expression of *SLC1A5* has been associated with poor prognosis for various types of cancer, e.g., in [29,30,31]. Another CpG site among our top 24 sites was located at this gene, namely cg02711608, which was also validated in the LBC. Hypomethylation of this specific site has been associated with type 2 diabetes [32]. In total, three of the 26 most significant sites in our study, two of the sites in the LASSO predictor, and three of the 58 CpG sites in [9] were located at *SLC1A5*, strongly indicating that hypomethylation of this region is associated with increased mortality.

The second most significant site, cg12627844, is located at the shore of a CpG Island in the 5’ region of the *VPS54* gene. Again, the presence of nearby histone mark enrichment, areas of DNA hypersensitivity, and transcription factor binding sites suggests that this is a regulatory region. This was not validated in the Lothian Birth Cohort and the information was not available in the Swedish dataset.

The third site in Table 2, cg05339037, was validated in the LBC and supported by SATSA. It is located to a shelf in the vicinity of a probable non-coding RNA gene of the miR-3074 gene family. The region is characterized by a high level of enrichment of the H3K27Ac histone mark, as well as DNase hypersensitivity, and numerous possible transcription factor recognition sites. This all points to the presence of a promoter.

In addition, the seventh site in Table 2, cg10589813, replicates in LBC and is supported by SATSA. It is located in the south shore of a CpG island, which lies near the *CEBPB* gene, encoding an important transcription factor involved in the regulation of the expression of genes involved in immune and inflammatory responses [33,34,35].

The 18th site, cg11339912, was also confirmed by the LBC. It is located at an intron of the *SH3RF2* gene. The gene is highly expressed in cancer tissue [36]. We did not encounter any relevant information on cg17087741, cg15871086, or cg02657160.

We also constructed a predictor for mortality. It showed good performance on the Danish dataset, and also under cross-validation and when correcting for at least genetic effects by MZ intra-pair comparison. It did not seem to lose predictive power during the follow-up period. However, it was not possible to validate it in any of the two independent datasets. This may be because the predictor is sensitive to latent cohort effects (see below) and the various steps taken in the data preprocessing phase. If a predictor is going to be used for predictions across samples in the future, it should come with a manual for data preprocessing and batch effect removal. Alternatively, one might take an approach that is more robust to noise, as in [9]. Including more sites in the predictor, as is done for the age predictors of [5,6], might also make it more robust against noise within a single site and account for more possible causes of death.

While our results were confirmed by the Lothian Birth Cohort to the degree that one might expect, we had problems validating the predictor in the Swedish twin data. This is surprising, as the two studies are very similar, both using samples of Scandinavian twins of comparable size, age span, and follow-up time. Again, this points to the problem of comparing methylation data across different studies, e.g., different preprocessing strategies (see also discussion below on the phenotype in question).

We were able to confirm many of the findings from [9]. This study benefits from a large sample size of twin pairs and an independent dataset used for validation. Moreover, a long list of confounders is controlled for in the validation phase. On the other hand, adjusting for confounders risks overlooking CpGs that e.g., mediate the effect of lifestyle factors or cause preexisting diseases. We did not control for such confounders in the present study. However, the finding that the identified sites to a large degree persist in the intra-pair comparison test, and are vulnerable to only non-shared non-genetic confounders, is highly indicative of an association. The intra-pair comparison is not feasible for the all the EWAS but adds validity to the candidate findings.

The sites found in [10,11] were not as convincingly validated in our dataset. In both cases, this may be due to low power in those studies (36 and 79 deaths, respectively) or differences in the study population. The individuals in [10] were all nonagenarians (aged 90+), and mechanisms causing deaths in very old people might be different from those present in our sample population. Moreover, both studies have much shorter follow-up times. It is possible that sites predicting mortality within the near future are different from those predicting long-term survival.

Finally, in contrast to EWAS on age-dependent epigenetic changes that reported high replication rates across samples [37], EWAS on mortality deal with an outcome variable (i.e., death) that is subject to more uncertainty than an individual age. Low overlap across studies can be expected due to power limitations across studies. A systematic meta-analysis or a combined analysis would be warranted to justify findings across studies.

Although deviations between chronological and epigenetic age have been observed to predict all-cause mortality [7,8], the CpG sites defining the methylation ages by Horvath and Hannum were generally not strongly associated with mortality in this study. Similar observations were made in previous studies of methylation and mortality (see the discussion sections in [9,10,11]). A possible explanation may be that sites are chosen for epigenetic clocks because of rather stable age-related changes in methylation independent of diseases, life-style factors, and individual aging patterns. The present contribution may be seen as an initial step among many in explaining systematic patterns of genetic and epigenetic causes that exist [38] for the phenotype of age at death, which is subject to considerable uncertainty.

## Figures and Tables

**Table 1:** Characteristics of the sample population. Cardiovascular diseases include apoplexy and age-related cardiovascular weakness.

Dataset	BWD	LSADT	MADT	Total
*N*	150	238	482	870
Women, *N* (%)	74 (49%)	156 (76%)	218 (46%)	448
Deaths, *N* (%)	11 (7.3%)	213 (89%)	34 (7.0%)	258
Age at blood sample	57–74	73–90	55–79	55–90
Year of blood sample	2009–2010	1996–1997	2008–2011
Follow-up year	2016	2016	2016	
Cause of death:				
Cancer	1	35	4	40
Cardiovascular disease	0	72	2	74
Respiratory causes	1	18	1	20

BWT: a study of birth weight-discordant twins; LSADT: Longitudinal Study of Aging Danish Twins; MADT: Middle Aged Danish Twins Study.

**Table 2:** The 24 CpG sites with $p<10^{-7}$ in the univariate analysis.

CpG Site	HR	*p*	FDR	Chromosome	Gene Name	MZ Correlation
cg07626482 ${}^{†}$	0.64	3.21l$\;\times \;10^{-12}$	1.42$\;\times \;10^{-6}$	19	*SLC1A5*	0.43 ***
cg12627844	0.69	5.20$\;\times \;10^{-9}$	1.15$\;\times \;10^{-3}$	2	*VPS54*	0.44 ***
cg05339037 ${}^{†}$	0.77	1.18$\;\times \;10^{-8}$	1.31$\;\times \;10^{-3}$	19		0.44 ***
cg03487027	0.73	1.40$\;\times \;10^{-8}$	1.31$\;\times \;10^{-3}$	10	*ZNF503*	0.51 ***
cg20162822	0.78	1.81$\;\times \;10^{-8}$	1.31$\;\times \;10^{-3}$	17	*SERPINF2*	−0.05
cg25121437	0.73	2.04$\;\times \;10^{-8}$	1.31$\;\times \;10^{-3}$	11	*FEZ1*	0.29 ***
cg10589813 ${}^{†}$	0.71	2.76$\;\times \;10^{-8}$	1.31$\;\times \;10^{-3}$	20		0.41 ***
cg08627624 ${}^{†}$	0.69	2.85$\;\times \;10^{-8}$	1.31$\;\times \;10^{-3}$	10		−0.04
cg06598544	0.78	3.13$\;\times \;10^{-8}$	1.31$\;\times \;10^{-3}$	20	*COL9A3*	0.31 ***
cg23591302 **	0.74	3.14$\;\times \;10^{-8}$	1.31$\;\times \;10^{-3}$	12	*PRICKLE1*	0.15 ***
cg13580749 ${}^{†}$	0.72	3.25$\;\times \;10^{-8}$	1.31$\;\times \;10^{-3}$	9		0.18 ***
cg15013177 *${}^{,}$**${}^{,†}$	0.77	3.98$\;\times \;10^{-8}$	1.31$\;\times \;10^{-3}$	3	*CNTN6*	0.12 ***
cg17087741 **${}^{,†}$	0.71	4.16$\;\times \;10^{-8}$	1.31$\;\times \;10^{-3}$	2		0.45 ***
cg04716447 **${}^{,†}$	1.42	4.16$\;\times \;10^{-8}$	1.31$\;\times \;10^{-3}$	12		0.18 ***
cg05232694 ${}^{†}$	0.72	4.64$\;\times \;10^{-8}$	1.33$\;\times \;10^{-3}$	20		0.31 ***
cg06172950 ${}^{†}$	0.74	4.81$\;\times \;10^{-8}$	1.33$\;\times \;10^{-3}$	13	*COG3*	0.27 ***
cg26186132 ${}^{†}$	0.71	5.66$\;\times \;10^{-8}$	1.41$\;\times \;10^{-3}$	6	*C6orf147*	−0.07
cg11339912	0.72	5.74$\;\times \;10^{-8}$	1.41$\;\times \;10^{-3}$	5	*SH3RF2*	0.39 ***
cg24750887 ${}^{†}$	1.42	6.22$\;\times \;10^{-8}$	1.44$\;\times \;10^{-3}$	4	*HERC3*	0.05
cg15763258 ${}^{†}$	0.73	6.52$\;\times \;10^{-8}$	1.44$\;\times \;10^{-3}$	11	*FLI1*	0.36 ***
cg15871086 ${}^{†}$	0.74	7.65$\;\times \;10^{-8}$	1.61$\;\times \;10^{-3}$	18		0.36 ***
cg02657160 ${}^{†}$	0.70	8.81$\;\times \;10^{-8}$	1.72$\;\times \;10^{-3}$	3	*CPOX*	0.18 ***
cg02711608 **${}^{,†}$	0.69	8.99$\;\times \;10^{-8}$	1.72$\;\times \;10^{-3}$	19	SLC1A5	0.48 ***
cg17459949 ${}^{†}$	0.73	9.79$\;\times \;10^{-8}$	1.80$\;\times \;10^{-3}$	10		−0.06

FDR: false discovery rate. * Polymorphic site according to [16]. ** Single nucleotide polymorphism (SNP) within-probe binding region (according to annotation file). *** Significant at the 0.05 level. † A significant hazard ratio (HR) at 0.05 level in intra-pair comparison.

**Table 3:** Hazard ratio and association *p*-value for the top 24 sites in a Cox frailty model adjusted for sex, age, and twin structure in the discovery and validation datasets. Five CpGs were not available in the Swedish data.

CpG Site	Danish Twins		Lothian Birth Cohort		Swedish Twins	
	HR	*p*	HR	*p*	HR	*p*
cg07626482	0.64	3.21$\;\times \;10^{-12}$	0.55	3.18$\;\times \;10^{-3}$	0.88	0.09
cg12627844	0.69	5.20$\;\times \;10^{-9}$	0.94	0.53	-	-
cg05339037	0.77	1.18$\;\times \;10^{-8}$	0.42	1.70$\;\times \;10^{-4}$	0.85	0.02
cg03487027	0.73	1.40$\;\times \;10^{-8}$	1.09	4.81$\;\times \;10^{-3}$	0.99	0.87
cg20162822	0.78	1.81$\;\times \;10^{-8}$	0.96	0.64	1.08	0.31
cg25121437	0.73	2.04$\;\times \;10^{-8}$	0.91	0.48	1.07	0.30
cg10589813	0.71	2.76$\;\times \;10^{-8}$	0.56	2.71$\;\times \;10^{-3}$	0.85	0.03
cg08627624	0.69	2.85$\;\times \;10^{-8}$	1.08	0.48	-	-
cg06598544	0.78	3.13$\;\times \;10^{-8}$	1.06	0.13	1.04	0.57
cg23591302	0.74	3.14$\;\times \;10^{-8}$	1.05	0.11	-	-
cg13580749	0.72	3.25$\;\times \;10^{-8}$	1.00	1.00	-	-
cg15013177	0.77	3.98$\;\times \;10^{-8}$	1.09	0.42	0.97	0.66
cg17087741	0.71	4.16$\;\times \;10^{-8}$	0.57	3.22$\;\times \;10^{-7}$	-	-
cg04716447	1.42	4.16$\;\times \;10^{-8}$	1.37	0.15	1.06	0.47
cg05232694	0.72	4.64$\;\times \;10^{-8}$	0.86	0.07	0.90	0.17
cg06172950	0.74	4.81$\;\times \;10^{-8}$	0.77	0.034	0.98	0.75
cg26186132	0.71	5.66$\;\times \;10^{-8}$	1.18	0.33	1.10	0.17
cg11339912	0.72	5.74$\;\times \;10^{-8}$	0.65	2.27$\;\times \;10^{-3}$	0.91	0.18
cg24750887	1.42	6.22$\;\times \;10^{-8}$	1.05	0.52	1.02	0.82
cg15763258	0.73	6.52$\;\times \;10^{-8}$	0.70	0.03	0.97	0.69
cg15871086	0.74	7.65$\;\times \;10^{-8}$	0.66	6.82$\;\times \;10^{-3}$	0.97	0.71
cg02657160	0.70	8.81$\;\times \;10^{-8}$	1.01	0.93	0.90	0.13
cg02711608	0.69	8.99$\;\times \;10^{-8}$	0.53	5.30$\;\times \;10^{-5}$	0.99	0.94
cg17459949	0.73	9.79$\;\times \;10^{-8}$	1.07	0.11	0.97	0.68

**Table 4:** The variables chosen by the least absolute shrinkage and selection operator (LASSO) for the mortality predictor. The second column gives the coefficients for the linear predictor. All methylation values are normalized to have a standard deviation of 1.

Covariate	Coefficient **	SE **	p **	Chromosome	Gene Name	MZ Correlation
cg02537149	0.158	0.0664	0.0173	1	*LRRC41, UQCRH*	0.00
cg02691019	0.0906	0.0564	0.108	16		−0.07
cg04716447 *	0.308	0.0623	7.36$\;\times \;10^{-7}$	12		0.18 ***
cg05232694	−0.155	0.0686	0.0234	20		0.31 ***
cg07626482	−0.160	0.0820	0.0508	19	*SLC1A5*	0.43 ***
cg12880095	0.115	0.0621	0.0650	17		0.53 ***
cg14304264	0.140	0.0705	0.0467	15	*MCTP2*	0.00
cg17459949	−0.176	0.0640	5.95$\;\times \;10^{-3}$	10		−0.06
cg20164226 *	0.0579	0.0579	0.317	7		0.27 ***
cg21381949	−0.112	0.0592	0.0585	3	*LEPREL1*	0.12 ***
cg22304262	−0.273	0.0822	8.97$\;\times \;10^{-4}$	19	*SLC1A5*	0.38 ***
cg24750887	0.205	0.0663	1.96$\;\times \;10^{-3}$	4	*HERC3*	0.05
cg24967142	0.219	0.0669	1.04$\;\times \;10^{-3}$	12	*C12orf47, MAPKAPK5*	−0.08
cg26186132	−0.228	0.0688	9.11$\;\times \;10^{-4}$	6	*C6orf147*	−0.07
Sex (female)	−0.702	0.140	5.71$\;\times \;10^{-7}$			
Age (in years)	0.139	0.0165	<2$\;\times \;10^{-16}$			

* SNP within the probe-binding region. ** Coefficients, standard error, and *p*-value in a Cox model fitted with all 14 sites, sex, and, age. *** Significant at the 0.05 level.

## Supplementary Materials

The following are available online at http://www.mdpi.com/2073-4425/9/2/78/s1, Figure S1: Cross-validtaed Harrell’s C for the 14 CpG sites used for the mortality predictor in the discovery and validation datasets, Figure S2: Correlations between the 14 CpG sites entering the mortality predictor, Table S1: A list of the all 2806 CpG sites with FDR<0.05 in the present study including information from annotation file, Table S2: List of all 35 CpG sites dicovered in [9] and showing significance in the present study.
