# Supplementary figures and images for: DNA Methylation and All-Cause Mortality in Middle-Aged and Elderly Danish Twins
Source: Genes (Basel). 2018 Feb 8;9(2):78. doi: 10.3390/genes9020078 (PMC5852574; doi:10.3390/genes9020078)

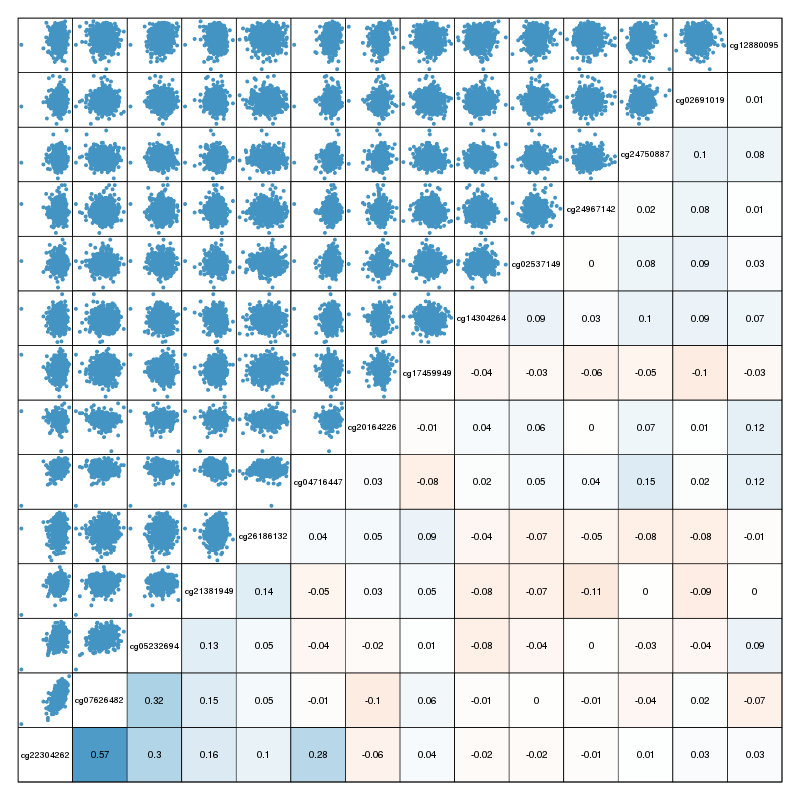

Supplement: Supplementary file 1 [file genes-09-00078-s001.zip › FigureS2.png]

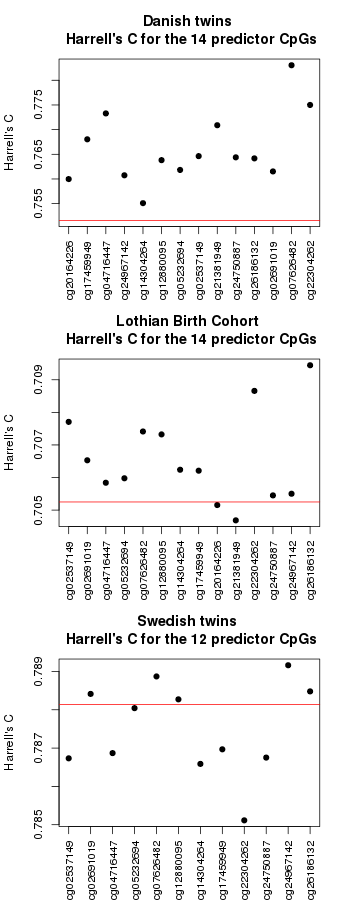

Supplement: Supplementary file 1 [file genes-09-00078-s001.zip › FigureS1.png]
